# Vasospasm and delayed cerebral ischemia after uneventful clipping of an unruptured intracranial aneurysm – a case report

**DOI:** 10.1186/s12883-019-1458-4

**Published:** 2019-09-16

**Authors:** Christin Campe, Jens Neumann, I. Erol Sandalcioglu, Ali Rashidi, Michael Luchtmann

**Affiliations:** 1 Department of Neurology, Medical Faculty, Otto von Guericke University Magdeburg, Leipziger Str. 44, 39120 Magdeburg, Germany; 2 Department of Neurosurgery, Medical Faculty, Otto von Guericke University Magdeburg, Leipziger Str. 44, 39120 Magdeburg, Germany

**Keywords:** Unruptured intracranial aneurysm, Vasospasm, Delayed cerebral ischemia

## Abstract

**Background:**

Due to improvements in both the quality and availability of intracranial imaging as well as the evolution of surgical and endovascular techniques during the last decade, the number of treatments of unruptured intracranial aneurysms (UIA) has increased steadily. However, it is not generally known that vasospasm can arise after an uneventful clipping.

**Case presentation:**

We present a case of a 69-year-old woman who suffered from vasospasm and delayed cerebral ischemia that occurred after an uneventful clipping of a UIA.

The aneurysm of the right middle cerebral artery was found incidentally via magnetic resonance imaging ordered after the patient complained of a short period of slight gait disturbances. To avoid a subarachnoid hemorrhage and consecutive complications like vasospasms, the patient elected microsurgical treatment. Clipping was managed by keyhole approach. Temporal clipping of the M1 was not necessary. After clip placement, appropriate flow in all distal segments was confirmed by indocyanine green video-angiography and micro-Doppler. The patient was discharged seven days after surgery without neurological deficits.

After 12 days, the patient developed at home a sudden drooping on the left side of the face. Upon admission to the emergency room, the patient was alert but slightly confused. Neurological examination revealed a left-sided hemiparesis and motor speech disorder. In contrast to the preoperative transfemoral catheter angiography, the subsequent right internal carotid angiogram showed clear signs of vasospasm along the M1 and M2 segments of the right middle cerebral artery.

Antithrombotic treatment with acetylsalicylic acid was begun. In accordance with guidelines for the treatment of subarachnoid hemorrhage and vasospasm, nimodipine was added. After 11 days the patient was discharged with no symptoms.

**Conclusion:**

Cerebral vasospasm as a cause of ischemic stroke after uneventful surgery for a UIA seems to be a rare but possibly underestimated etiology that demands particular attention with respect to providing appropriate treatment. In future, it may be prudent to perform follow-up transcranial ultrasonography testing after the clipping of a UIA, especially considering the availability of potentially neuroprotective medications like nimodipine.

## Background

An unruptured intracranial aneurysm (UIA) for the most part involves saccular weakening and a bulging of arterial walls at major branches of supra- and infratentorial brain arteries and can be found in almost 5% of the adult population [1]. In recent decades, due to improvements in the quality and availability of intracranial imaging, UIAs have been more frequently detected, which has led to uncertainties with respect to appropriate treatment [2]. The most severe consequence of an aneurysm is a rupture that results in a subarachnoid hemorrhage (SAH), a devastating disease with high morbidity and mortality, regardless of any geographical or ethnic considerations [3]. One of the most severe complications of SAH is delayed cerebral ischemia (DCI) due to cerebral vasospasm. Despite intense research and the discovery of several pathogenic mechanisms, the precise pathophysiology of the development of vasospasm and its appropriate treatment remain poorly understood [4]. DCIs account for about 15% of mortality and morbidity due to SAH [5]. In order to avoid SAH and corresponding complications like vasospasm, UIAs have received treatment that varies depending on certain specific circumstances (e.g. size, location, shape, and growth rate) [6]. Treatment options comprise endovascular as well as surgical techniques.

However, it is not generally known that DCI can arise after an uneventful clipping of a UIA, and therefore little attention has been paid to this possibility. Neuroprotective medication (e.g., nimodipine) seems to be beneficial in such cases, and thus identifying the underlying cause of the stroke is crucial to improve the outcome for these patients.

## Case presentation

A 69-year-old woman developed a sudden drooping on the left side of the face while having dinner with her family. Her daughter noticed slurred speech and alerted emergency medical services immediately. The patient was pre-announced to the stroke service by the responding emergency medical technician and immediately admitted to the emergency room. Her home medication consisted of pantoprazole only. Upon admission to the emergency room, the patient was alert but slightly confused. Further neurological examination revealed a left-sided hemiparesis and motor speech disorder. The remaining cranial nerves were unaffected. No sensory or coordinative dysfunctions were detected. Muscle stretch reflexes revealed no lateral differences, and plantar reflexes were normal (NIHSS score: 4 points). Shaved hair over the right temple exposed a well-healing, 10-cm-long recent wound. The patient reported having had brain surgery two weeks earlier, but upon further questioning denied a preceding trauma, infection, tumor disease, or cerebral bleeding.

**Fig. 1:**
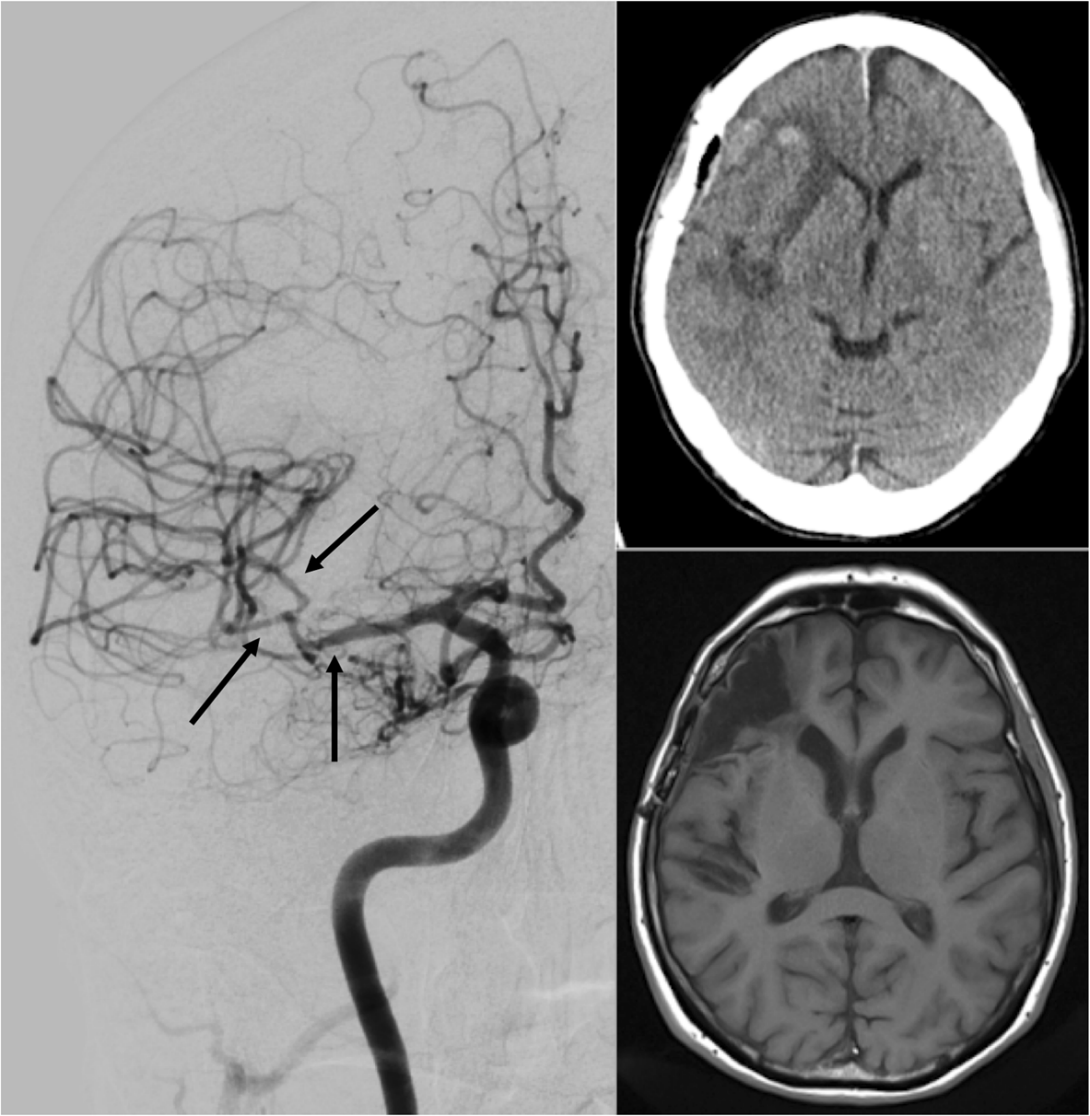
Postoperative imaging with proof of vasospasm of the M1 and M2 segments of the right MCA (black arrows), CT imaging at admission and MRI follow up after 6 months

The non-contrast computed tomography (CT) imaging revealed hypodense areas in the circulation of the middle cerebral artery (MCA) with territorial pattern (mainly pre-Rolandic, but also Rolandic, parietal, and insular branches), moderate swelling, and hemorrhagic transformation of the anterior portion (see Fig. 1). A vascular clip in projection on the middle cerebral artery was visible. There was no sign of a subarachnoid hemorrhage (SAH). The CT-angiography revealed no high-grade stenosis or vessel occlusion of the cerebral blood flow in the area of the right middle cerebral artery, even though the presence of a vascular clip reduced reliability of assessment. The cerebral duplex ultrasonography/transcranial Doppler sonography (TCD) showed, in contrast to the left side, markedly increased blood flow velocities in the right MCA with mean values up to 180 cm/s (V_max_ up to 300 cm/s), while the blood flow in all of the other cerebral arteries was undisturbed. The increased velocities were traceable along the entire M1 segment as well as in the M2 segments of the right MCA. In contrast to the preoperative transfemoral catheter angiography (TFCA), the subsequent right internal carotid angiogram showed clear signs of vasospasm along the M1 and M2 segments of the right MCA (see Fig. 1). However, neither delayed cerebral blood flow nor hypoperfusion were found. A vessel narrowing with consecutive stenosis due to a suboptimally placed clip was ruled out.

**Fig. 2:**
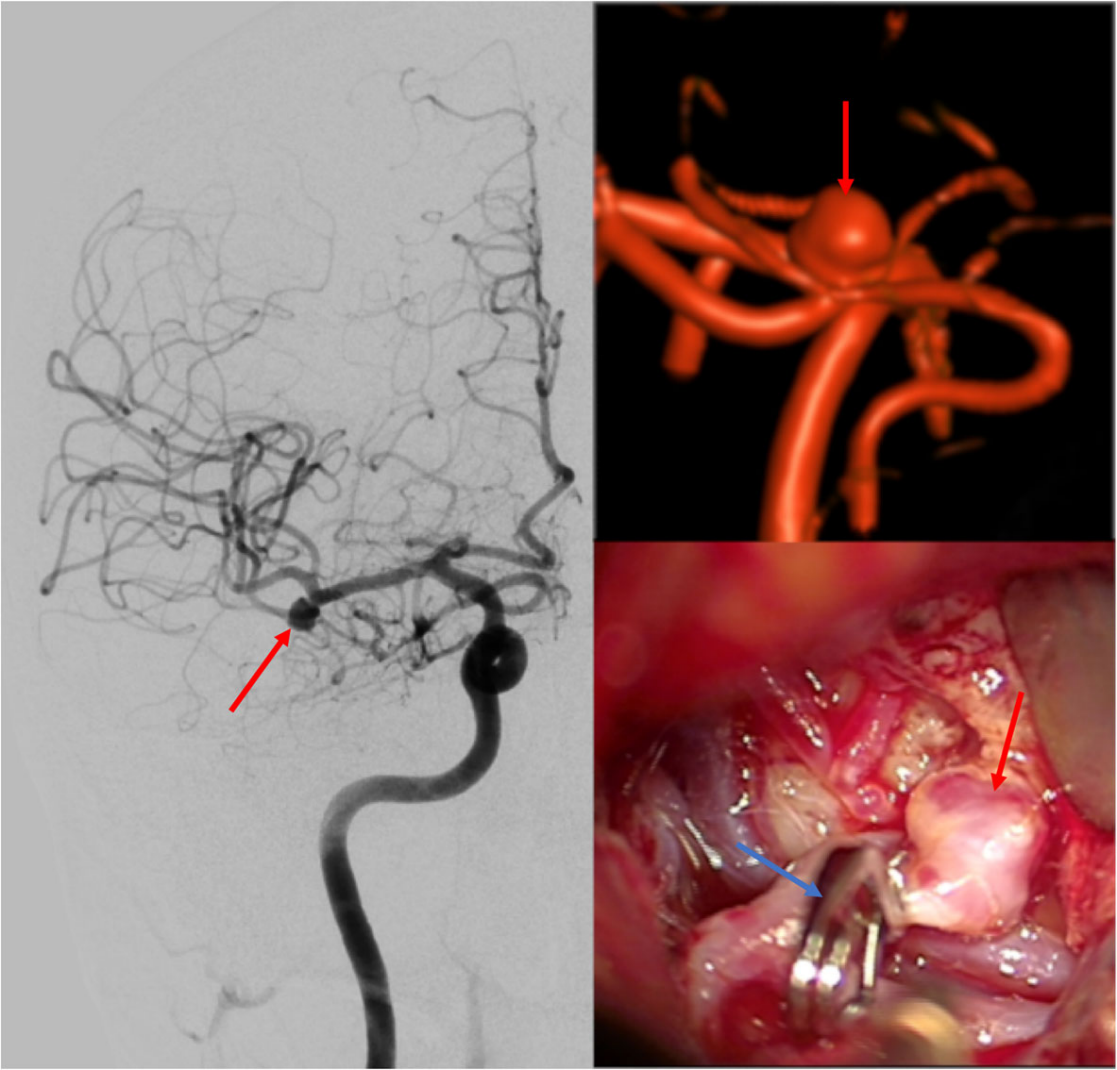
Preoperative imaging of the untreated MCA aneurysm (red arrows) and intraoperative view after proper placement of the clip (blue arrows)

The patient’s recent medical history included the microsurgical treatment of a right-sided MCA aneurysm 12 days prior. The patient had never experienced any episodes of uncommon or severe headaches. The unruptured intracranial aneurysm (UIA) was found incidentally via magnetic resonance imaging ordered after the patient complained of a short period of slight gait disturbances. To avoid an SAH and consecutive complications like vasospasms, the patient elected surgical treatment (see Fig. 2). Endovascular management was not feasible due to the configuration of the aneurysm. The review of the operative report and the medical discharge letter attested to an uneventful perioperative course. Clipping was managed by keyhole approach. A craniotomy 30 mm in diameter was performed over the right Sylvian fissure. The aneurysm was dissected after securing proximal control of the distal M1 segment of the right MCA. Temporal clipping of the M1 was not necessary. After clip placement, appropriate flow in all distal segments was confirmed by indocyanine green video-angiography and micro-Doppler. The postoperative imaging showed no sign of decreased cerebral blood flow. The patient was discharged seven days after surgery without neurological deficits. No other vascular diseases were known.

After admission antithrombotic treatment with acetylsalicylic acid was begun. In accordance with guidelines for the treatment of subarachnoid hemorrhage and vasospasm, nimodipine was added. Periodically performed transcranial duplex sonography showed a further increase of blood flow velocity in the MCA and its branches for four days before a continuous decrease and normalization of flow velocity was observed. Treatment with nimodipine was continued for an additional two weeks. Within this time the symptoms disappeared completely. The patient made a full recovery, which is remarkable in such a major stroke. After 11 days the woman was discharged with no symptoms.

## Discussion and conclusions

It is not commonly known that cerebral vasospasm may occur after surgical clipping of a UIA. Here we present the case of a patient developing DCI due to vasospasm after an uneventful surgical occlusion of an unruptured MCA aneurysm. Only a few cases of DCI after clipping of a UIA have been reported in the last few decades [7]. Among these, the onset of symptoms varied considerably, ranging from 5 to 28 days after surgery. In contrast, vasospasm onset after SAH typically occurs within 14 days. Risk factors that are associated with DCI are unknown. It is assumed that temporal clipping and the application of two or more clips increase the risk for the development of vasospasm. In the present case, only one clip had been applied, and without the necessity of performing a temporary clipping.

Despite its high incidence, the precise pathophysiological mechanisms of vasospasm following SAH are still not fully understood. It is therefore not surprising that the pathophysiology of vasospasm following surgical treatment of UIA is also not understood. Some intriguing mechanisms are assumed to contribute. DeLong has proposed that erythrocytes’ breakdown products within the aneurysm sac may account for the development of vasospasm [8]. That hypothesis was derived from the observation that resection, rather than the simple occlusion of the aneurysm, was related to a decreased incidence of vasospasm in patients suffering from SAH. A diffusion of blood breakdown products through the arterial wall is quite speculative. However, this slow process would account for the delay of occurrence of vasospasm. Another potential pathophysiological mechanism for vasospasm associated with an unruptured and untreated aneurysm has been reported by Friedman and colleagues [9]. An unruptured aneurysm underwent symptomatic changes of size within a few days. It was assumed that the property to produce vasorelaxant lipoproteins was impaired in the adjacent endothelium. This hypothesis would support the mechanical theory, which proposes that instrumental manipulation involving the arterial endothelial layer (e.g., due to temporal clipping) at the major branches of the cerebral arteries may lead to impaired cerebrovascular reactivity [10]. Contemporary theories presume that the source of vasospasm might be the cerebral arteries themselves. Particular attention has been directed at the adventitial network of nerves that is believed to initiate spasmogenic mechanisms rather than endothelial factors [11].

Because the incidence of vasospasm and DCI following the clipping of a UIA seems to be low and the pathophysiology is not fully understood, no treatment guidelines are available. However, in the present case, the treatment was derived from the guidelines for management of an SAH [12]. For that reason, nimodipine was administered orally with a daily dosage of 360 mg. Transcranial ultrasonography was used to monitor the progression and regression of the vasospasm.

According to the few case reports available that are similar to the present case, most patients made a full recovery. Some patients, however, were reported to have had a poor outcome.

In our opinion, a probabilistic approach to clinical decision-making is always beneficial. If it looks like a duck, swims like a duck, and quacks like a duck, then it probably is a duck—but not always. Cerebral vasospasm is not the most common etiology in patients suffering a stroke, particularly in elderly patients with UIA. In the present case, all available diagnostic means were exploited to determine the cause of the stroke, including cerebral duplex ultrasonography and TFCA. In addition to the classic presentation of a spontaneous aneurysmal SAH, there are other factors that may contribute to the development of transient vessel narrowing. The similar clinical presentation of reversible cerebral vasoconstrictive syndrome (RCVS) is also associated with transient increased blood flow velocity, but without evidence of an SAH or an aneurysm. RCVS most commonly occurs because of vasoactive substances or during the postpartum period, but a cerebral venous sinus thrombosis or a traumatic brain injury may also cause increased cerebral blood flow velocity. In this case, typical angiographic signs of vasospasm and an exhaustive review of the medical history of the patient led to the proper diagnosis.

This patient with a surgically treated UIA developed vasospasm in the absence of an SAH. It is crucial to discern that the absence of a subarachnoid hemorrhage in patients with UIA does not preclude the presence of cerebral vasospasms. The incidence of vasospasm and DCI after a clipping of the UIA is completely unknown. There are probably several reasons for this lack. On the one hand, the clinical staff often does not know that DCI can arise after an uneventful clipping, and as a result, little attention is paid to this possibility. The latency of vasospasm onset for up to three weeks postoperative is another pitfall to consider. In one case, the report of onset of symptoms was 28 days after surgery [13]. By that time, after an uneventful clipping of a UIA, most patients have usually been discharged as free of neurological symptoms. Thus, it may be difficult to recognize a causal relationship with such a time gap. On the other hand, the evolution of surgical techniques in the last decade has opened up the possibility of performing relatively low-risk surgeries in the elderly. Therefore, coinciding events such as an ischemic stroke are not unlikely.

To further stratify the risk of clinically relevant vasospasm in the management of UIA treatment, it may be beneficial to perform pre- and postoperative transcranial duplex sonography / TCD, especially considering the availability of the neuroprotective calcium channel blocker nimodipine. The resulting findings on the incidence of subclinical and clinical vasospasms after the clipping of a UIA would be of significance in the prevention of stroke.

In conclusion, cerebral vasospasm as a cause of ischemic stroke after uneventful surgery for a UIA seems to be a rare but possibly underestimated etiology that demands particular attention with respect to providing appropriate treatment. In future, it may be prudent to perform follow-up transcranial ultrasonography testing after the clipping of a UIA, especially considering the availability of potentially neuroprotective medications like nimodipine.

## Data Availability

Not applicable. Data sharing is not applicable to this article as no datasets were generated or analyzed.

## Abbreviations

CT: Computed tomography
DCI: Delayed cerebral ischemia
MCA: Middle cerebral artery
RCVS: Reversible cerebral vasoconstrictive syndrome
SAH: Subarachnoid hemorrhage
TCD: Transcranial Doppler sonography
TFCA: Transfemoral catheter angiography
UIA: Unruptured intracranial aneurysm
